# Preservation of Food

**Published:** 1859-09

**Authors:** 


### PRESERVATION OF FOOD.

It is the common air which sustains the life of all that breathes or grows, but when breath and growth cease, that same air is the agent of destruction, and reduces all to ashes and dust. But in proportion as we can successfully exclude the common air from anything which has parted with life, whether animal or vegetable, it may be indefinitely preserved. Meat begins to decompose after a few hours exposure to a warm sun, but human ingenuity has devised means for keeping it fresh for weeks, and months, and years even in warm climates. Milk begins to decompose within an hour after it is drawn from the cow, but the genius of Gail Borden has laid New York under contribution by supplying it with a concentrated article which maintains its freshness for weeks, and even months. This gentleman is also the unacknowledged instrument in the preservation of Dr. Kane and his men, on their mission of humanity for Sir John Franklin, for they were rescued from imminent starvation by food prepared by a process of his own devising. It is not known whether he is still pushing his experiments in that direction, but it is very certain if government had extended to him a modicum of the moral countenance and material aid bestowed on experiments in the construction of murderous fire arms, humanity might have been benefited to an incalculably greater extent.

The secret of the success of the self-sealing cans in preserving fruits, berries and vegetables, lies in the perfection and handiness with which common air is excluded. Yet, after all, they are but a questionable improvement of the plan of our grandmothers, who used to fill common bottles with the desired fruit, then pouring in hot syrup to fill up the interstices, the cork was put in loosely, and the whole placed in boiling water for a minute or two; the cork was then driven home, the bottles placed neck downwards in a trench, in the earth, in the cellar, covered over and let alone for use in after months or years.

Sir John Ross states that a tin case of preserved beef was landed from the "Fury" in August, 1825, and taken by him in July, 1833. This case he presented to a friend several years later, and in April 1859, it was opened at a bachelor's party.

"Along with the entrees came the contents of the tin case of boiled beef, which proved to be as sweet and fresh, and containing as much nourishment as it formerly did, when carried to the Arctic regions in the unfortunate Fury, in 1825." Taking this statement as true, and there is no reason to doubt it, food carefully put up, can be preserved thirty-four years.

The old time plan of bottles buried, is safer and better than any other for the preservation of fruits, and berries for domestic use. Tin cans, and glazed crockery are liable to be acted on by the acid of fruits and berries, so as to produce poisonous effects, bnt glass is indestructible by such chemical agents, it is cheap, and can be had anywhere; besides, it is more readily and more perfectly cleaned, and the mode of preparation is simple and easy.

Those who prefer to use the patent self-sealing cans or jars, should give the glass ones the preference; next, the glazed stone ware; and tin, last. It would greatly promote the health and comfort of families, if bushels of fruits and berries, and tomatoes were put up for winter use, instead of quarts and gallons; not in the costly and laborious method of old time "preserving," but on the more simple plan of the present day, by which they can be preserved in their nearly natural state, little or no sweetening being required.

